# A Qualitative Study of Airborne Minerals and Associated Organic Compounds in Southeast of Cairo, Egypt

**DOI:** 10.3390/ijerph15040568

**Published:** 2018-03-21

**Authors:** Kamal T. Hindy, Ashraf R. Baghdady, Fares M. Howari, Ahmed S. Abdelmaksoud

**Affiliations:** 1 Air Pollution Research Department, Environmental Research Division, National Research Center, Giza 12622, Egypt; kamalhindy@yahoo.com (K.T.H.); asabdelmaksoud@yahoo.com (A.S.A.); 2 Department of Geology, Faculty of Science, Ain-Shams University, Cairo 1156, Egypt; arbaghdady@sci.asu.edu.eg; 3 College of Natural and Health Sciences, Zayed University, Abu Dhabi 144534, UAE; 4 Environmental Science Department, Faculty of Meteorology, Environment and Arid Land Agriculture, King Abdulaziz University, Jeddah 21589, Saudi Arabia

**Keywords:** mineralogy, dust fall, polarizing microscope, infra-red spectroscopy, X-ray diffraction, organic compounds

## Abstract

This study is concerned with the identification of the mineralogical composition of dust fall samples collected from southeast of Cairo, Egypt. The mineralogical identification was conducted by means of the polarizing microscope, infra-red spectroscopy (IR), and X-ray diffraction (XRD). The relationship between the mineralogical composition of dust fall samples and 10 rock samples from the surrounding terrains were investigated. The major mineralogical species existing in the atmosphere of the study area are: carbonates mainly in the form of calcite in addition to the appearance of the dolomite form in traces overall the study area, but with considerable observation in the southern region; quartz which is less than calcite in its abundance; sulphates in the form of gypsum which may also be present as traces in the anhydrite form. Trace constitution of feldspars; clay minerals in the form of kaolinite, illite, and montimorillonite; and halite are also observable in the same samples. Organic compounds are present in the atmosphere of the area mainly as alkanes with presence of traces of phosphines. This study qualitatively shows the mineralogy of air particulate over rock processing area and the obtained results indicates that the main pollution source in the study area is the industrial activities with minor contribution of the natural sources, especially erosion and dust carried by winds from the surrounding terrains Cairo in the southern direction. This study provides useful results for the contribution of rock processing activities to the mineral composition of atmospheric particulates.

## 1. Introduction

Atmospheric particulate has been studied throughout the world, but with little emphasis on Egypt [1,2]. Scanning electron microscopy (SEM) with energy dispersive X-rays (EDX) have been used to study particles in morphological and chemical terms [3,4,5,6,7,8]. However, there are scattered literature on mineralogical analyses of atmospheric particles [9,10,11,12,13]. In certain health related cases, the mineralogy of atmospheric particles could be more useful than only knowing the elemental composition [14]. Dust aerosol has several environmental impacts, it influences climate through forcing by scattering and absorbing incoming solar radiation and by nucleating clouds [15,16]. Mineral dust aerosol influences also biogeochemical cycles as iron-containing minerals provide an important nutrient for ocean life [17]. More importantly, mineral dust aerosol in the respirable size range is deleterious to human health [18,19]. 

The study area is located to the southeast of Cairo and has the largest number of factories for manufacturing marble and granite in Egypt. This area was first occupied by quarrymen quarrying the limestone in the hills and mountains of Katameya and Tura. Over time, interested investors in this area had granted about 10,000 m^2^ to build up their stone-processing factories. Quarrymen who started to work in limestone processing had changed their factories to process marble and granite, which are more profitable. Marble and granite factories are concentrated in the study area, which has a main road divided into two streets where marble and granite factories are closely located next to one another. The study area has the highest number of marble and granite factories in Egypt reaching around 400 factories, constituting 60–70% of marble and granite factories found in the whole country which has a very diverse and complex geology [20].

This study is concerned with identification of the mineralogical composition of dust fall samples collected from the Shaq El-Teeban area located to the southeast of Cairo and selected 10 rock samples from the rocks commonly processed in this area by means of the polarizing microscope, infra-red spectroscopy, and X-ray diffraction (XRD). The relationship between the mineralogical composition of dust fall samples and that of the ten rock samples analyzed is illustrated.

## 2. Materials and Methods

**Figure 1:**
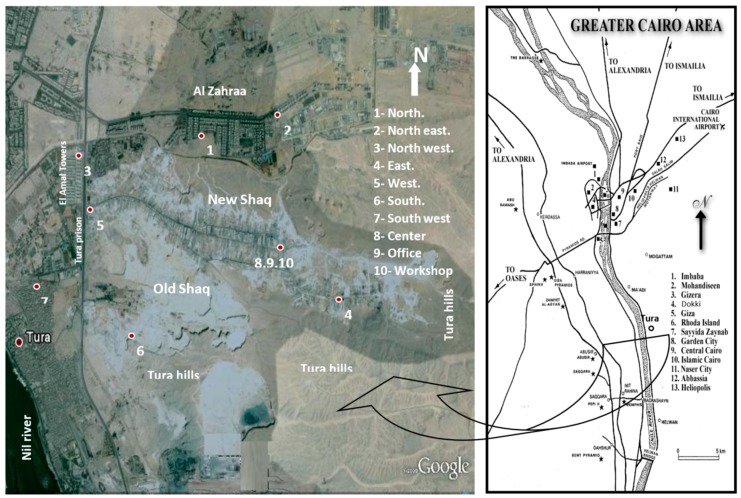
Location of the study area and observation sites.

Dust fall samples were collected monthly from 10 locations in the study area (north, northeast, northwest, east, west, south, southwest, center, an office, and a workshop) as shown in Figure 1. The sampling of the area took about 24 months to capture the temporal variations in the samples. The deposit gauges used for collection of samples are of the same type previously used in similar studies [21,22,23]. Although the present study adopted similar standard techniques to collect the samples, the analyses and analytical tools (e.g., SEM, and EDX) used in the present study were not performed in the earlier studies. 

Monthly collected samples from each location were composited to give an annual sample, the total number of dust fall samples investigated is 20. The examined rock samples are Fass Galala, Plain Galala, Treasta, Local Selvia, Golden Sinai, limestone, Hashmi stone, Red Royal granite, Gandola granite, and Indian Green marble which are the most common kinds of rocks processed in the area under investigation. The selected rocks in this study represent from the geological point of view three main rock types. These are: limestone, serpentinite, and granite. The limestone includes all carbonate rocks such as Fass Galala, Plain Galala, Tresta, Local Silivia, Golden Sinai, limestone, and Hashmi stone. Serpentinite is represented by Indian Green marble and granite is represented by Red Royal granite and Gandola granite.

### 2.1. Polarizing Microscope

The 20 dust fall samples and the 10 rock slide samples were petrographically described using a polarizing microscope. Each slide was prepared by sprinkling a few grains of the sample on a heated slide. They were stirred with a glass rod to scatter them within the adhesive material. After cooking of these materials, the upper surface of the cooled slides was ground flat and polished before its cover was applied.

### 2.2. Infrared Spectroscopy

The inorganic groups, the organic groups and the minerals constituting the dust fall and rock samples were identified according to [21,22,23]. Samples were prepared for spectroscopic examination in the form of a powder distributed through a matrix of alkali halide (potassium bromide of infrared quality). All the spectra obtained were recorded on a Fourier Transform (FT/IR-6100) infrared spectrophotometer, manufactured by JASCO, Japan. The frequency range used (4000–400 cm^−1^) covers the spectra of the detected compounds listed in Table 1 and Table 2.

### 2.3. X-ray Diffraction Analysis

A portion from each dust and rock samples was completely ground to 200 mesh and then analyzed without any chemical treatment. The diffraction peaks between 2° and 60° 2θ were recorded using a Philips X-ray diffractometer, model PW/1710, with Cu−Kα radiation (*λ* = 1.542 Å) at 40 Kv, 35 mA and scanning speed of 0.02°/S. The corresponding (dÅ) and the relative intensities (I/I°) were measured and compared with the standard American Society for Testing and Materials (ASTM) data and those published by the International Center for Diffraction Data (1995). 

## 3. Results and Discussion

### 3.1. Microscopic Examination

#### 3.1.1. Dust Fall Samples

**Figure 2:**
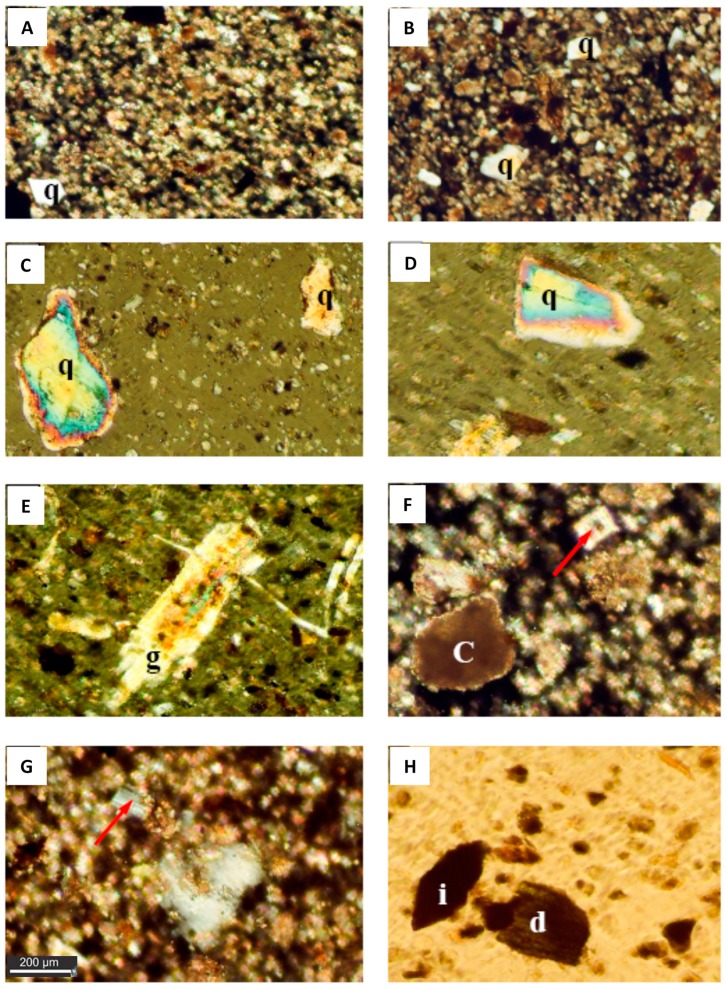
Photomicrographs showing the mineralogical composition of the dust fallout during the studyp. The background is composed of highly ferroginous microcrystalline calcite and clay minerals with scattered angular quartz (q) grains (Crossed Polar, 10X; image (**A**) and (**B**)); Images (**C**) and (**D**) show subangular to subrounded quartz (q) grains showing the evidence of wear and iron oxide inclusions. In these micrographs the gypsum crystal (arrow) in photo (**D**) and (**E**) (Crossed Polar, 10X) is well developed gypsum crystal; whereas the micrograph (g) shows iron inclusions (Crossed Polar, 10X); (**F**) zoned dolomite crystal (arrow) in a background of dolomitized calcite. Clay film (c) appears over calcite (Crossed Polar, 20X); (**G**) Plagioclase crystal (arrow) showing lamellar twining (Crossed Polar, 20X); (**H**) Green hornblende (d) and iron aggregate (i).

**Figure 3:**
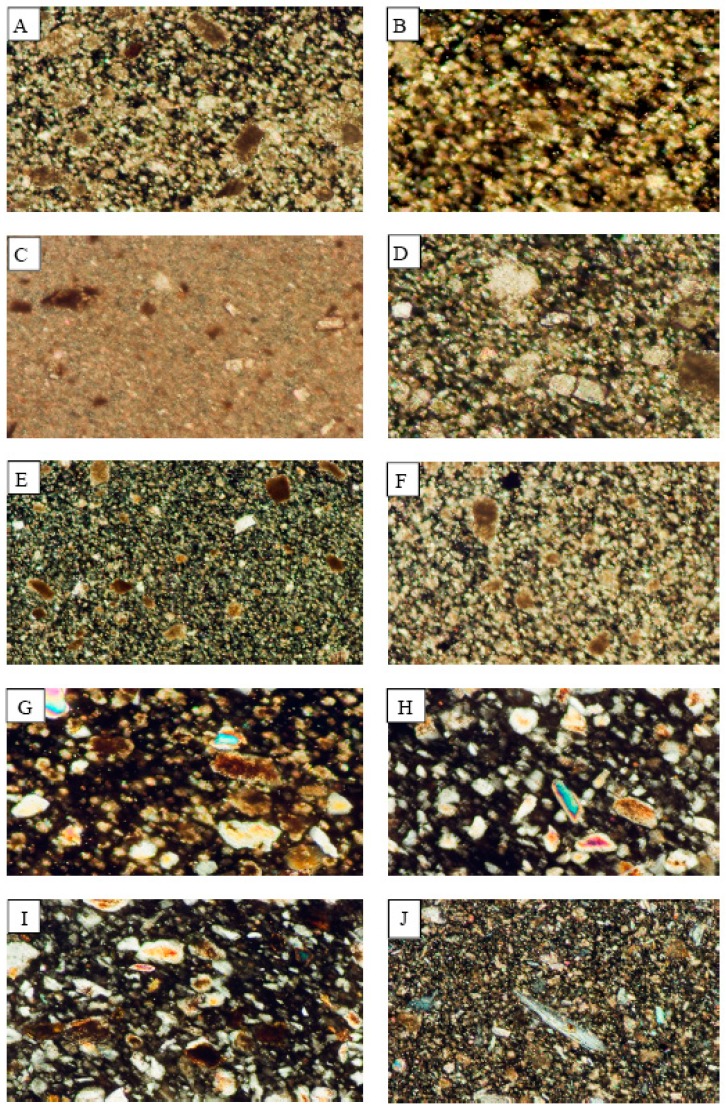
Photomicrographs showing the mineralogical composition of the rocks which are processed in the industrial sites of the study area. (**A**) Fass Galala; (**B**) Plain Galala; (**C**) Treasta; (**D**) Local Selvia; (**E**) Golden Sinai; (**F**) limestone; (**G**) Hashmi stone; (**H**) Red Royal granite; (**I**) Gandola granite; (**J**) Indian Green marble.

Microscopic examination of the solid mounts of dust fall grains reveals that the dust fall over the study area is mainly composed of calcite with subordinate amounts of quartz and gypsum (Figure 2 and Figure 3). Other minerals are rarely recorded and represented by clay minerals, dolomite, feldspars, and heavy minerals (plagioclase and hornblende).

(a) Calcite (CaCO_3_): It is the most abundant mineral in the dust fall over all observational sites. Calcite grains are commonly angular to subangular, microcrystalline and occasionally coarse crystalline.

(b) Quartz (SiO_2_): It is less abundant than calcite and recorded in all observational sites. These grains are silt to fine-sand-sized, subrounded to well rounded, mainly monocrystalline, and they commonly exhibit unit extinction. Most of the quartz grains contain inclusions of opaques and are coated with thin films of iron oxides. The latter are present as aggregates and/or fracture fillings. Quartz overgrowths have been recorded and in cases show signs of wearing.

(c) Gypsum (CaSO_4_·2H_2_O): It is recorded as minor constituent in dust fall over most of the observational sites. It is megacrystalline with some iron oxide inclusions.

(d) Clay minerals (Hydrated Aluminum Silicates): These are recorded in dust fall samples collected from all sites of investigation. However, their types are difficult to identify using the normal polarizing microscope.

(e) Dolomite (CaMg(CO_3_)_2_): Some grains of zoned dolomite and dolomitized calcite are observed in the dust fall collected from the eastern and southern sites.

(f) Plagioclase ((Na,Ca)(Si, Al)_4_O_8_): They are rarely recorded.

(g) Hornblende (Ca(Mg, Fe)_4_Al(Si_7_Al)O_22_(OH,F)_2_): The recorded hornblende is very rare and appears in the form of green grains.

#### 3.1.2. Rock Samples

The mineralogical composition of the examined carbonate rocks is limited to calcite and dolomite with rare quartz. Both the Red Royal Granite and Gandola Granite have the same composition which is represented by quartz, plagioclase, and hornblende. On the other side, Indian Green is represented by serpentinite which is mainly composed of serpentine mineral. The microscopic examination of the rock samples (Figure 3) reveals a great similarity between the mineralogical compositions of the dust fall and those of the rocks.

### 3.2. Infrared Spectra Interpretation

**Figure 4:**
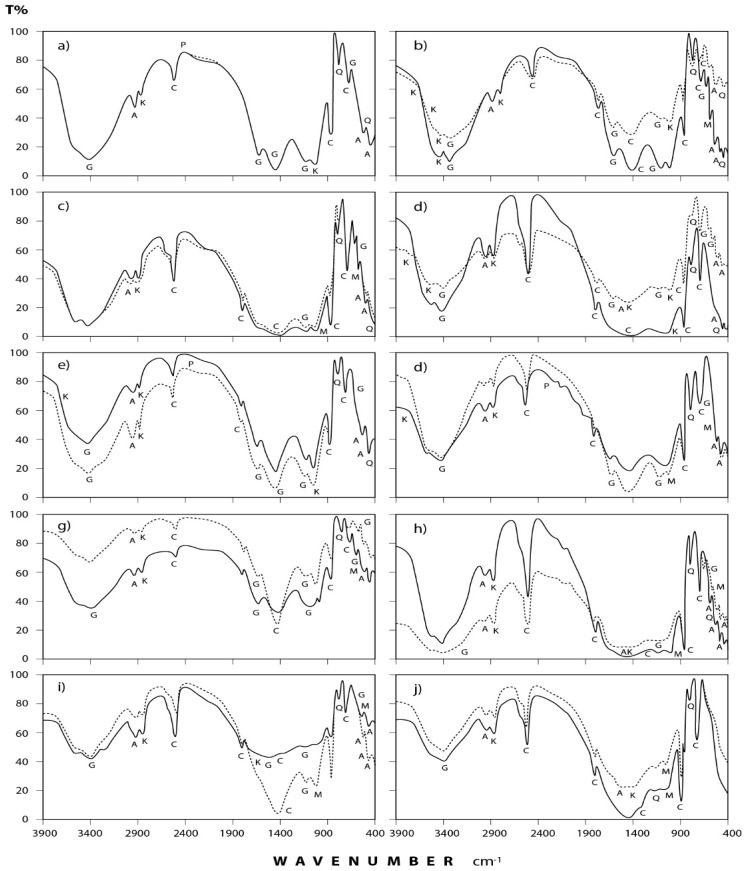
IR spectra of the minerals and organic species detected in annual dust fall samples at Shaq El−Teeban Industral Area during the two years of study. The normal lines represent the first year and bold lines represent the second year: (**a**) North location; (**b**) Northeast location; (**c**) Northwest location; (**d**) South location; (**e**) Southwest location; (**f**) East location; (**g**) West location; (**h**) Center location; (**i**) Office location; (**j**) Workshop location; A = albite; AK = alkane; C = calcite; G = gypsum; K = kaolinite; M = microcline; P = phosphine; Q = quartz.

**Figure 5:**
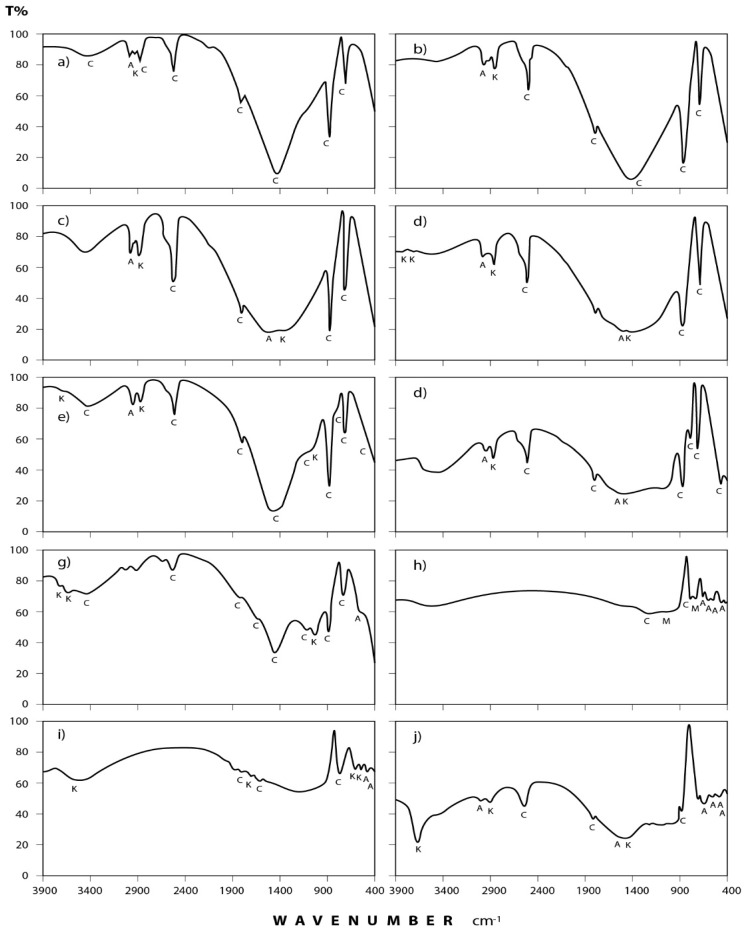
IR spectra of the minerals and organic species detected in stones commonly processed at Shaq El−Teeban Industral Area. (**a**) Fass Galala; (**b**) Plain Galala; (**c**) Treasta; (**d**) Local Selveia; (**e**) Golden Sinai; (**f**) limestone; (**g**) Hashmi stone; (**h**) Red Royal granite; (**i**) Gandola granite; (**j**) Indian Green marble. A = albite; AK = alkane; C = calcite; G = gypsum; K = kaolinite; M = microcline; Q = quartz.

Figure 4 and Figure 5 show the transmittance spectra of the analyzed dust fall and rock samples, respectively. The corresponding detected minerals and organic compounds are shown in the same figures. The principal identified mineralogical constituents are carbonates (calcite), sulphates (gypsum), clay minerals (kaolinite), quartz, feldspars (albite and microcline). Associated organic species are found in two forms: saturated aliphatic hydrocarbons (alkanes) and organic phosphorous compounds (phosphines). The characteristics of these minerals and compounds are discussed in the following subsections:

#### 3.2.1. Dust Fall Samples

(a) Carbonates: This group is present mostly in the form of calcium carbonate (calcite) which is identified through its spectral peaks at 2517, 1798, 1435, 876, and 712 cm^−1^ wave numbers [24,25,26]. Calcite appears in all spectra obtained for dust fall over the 10 sampling sites.

(b) Sulphates: Infrared spectroscopic analysis shows the presence of sulphates as hydrated calcium sulphate (gypsum). Its spectral peaks appear at 3410, 1626, 1142, 1114, and 670 cm^−1^ wave numbers [24,27,28]. These peaks are found in all the investigated samples except in workshop samples where single peak was detected at 3410 cm^−1^ and this may be attributed to the rocks which are usually processed in this workshop rarely containing gypsum.

(c) Clay minerals: Infrared spectra obtained for the dust fall in the study area indicate the presence of clay minerals in the form of kaolinite. This is shown through the diagnostic spectral peaks of the kaolinite which are present at 3677, 3613, 3547, and 1031 cm^−1^ wave numbers [24,28,29,30].

(d) Quartz: The presence of silicon dioxide as quartz is evident through examining the obtained spectra. Three spectral frequencies at 1163, 798, and 455 cm^−1^ wave numbers are characteristic of this mineral [28,31,32]. These are displayed by patterns of all the analyzed samples.

(e) Feldspars: Infrared spectroscopic analysis indicates the presence of two kinds of feldspar minerals in all of the dust fall samples—namely soda feldspar, represented by albite, and potash feldspar, represented by microcline. Spectral frequencies of albite appear at 529, 462, and 427 cm^−1^ wave numbers. Albite present in all collected samples with the exception of workshop samples. Microcline spectral frequencies appear at 1037 cm^−1^ and 585 cm^−1^ wave numbers [24,32] and were present in most of the analyzed samples. 

(f) Alkanes: Saturated aliphatic hydrocarbons containing methylene group of the general formula: C–CH_2_–C can be identified in all the analyzed samples at the wave numbers: 2976, 2927, 2871, and 1475 cm^−1^ [33,34]. 

(g) Phosphines: Investigated spectra show the presence of phosphines; PH_3_ (organic phosphorous compounds) in the analyzed samples. Spectral bands at the wave numbers 2440, 2379, and 1438 cm^−1^ were recorded in five locations (north, southwest, east, center, and workshop) of the study area [33,34].

#### 3.2.2. Rock Samples

(a) Carbonates: Examination of infrared spectra of the investigated rock samples indicates that they are mainly formed of calcite. This is the predominant component of the selected rocks for the present study. However, calcite is present in minor amounts in Gondola granite and it is nearly absent in Red Royal granite.

(b) Sulphates: The obtained infrared spectra show that gypsum is present in considerable amounts in Hashmi rocks and in minor amounts in Fass Galala, Golden Sinai, and Gandola granite, meanwhile it cannot be observed in other types of investigated rocks.

(c) Clay Minerals: As indicated from Infrared analysis, clay minerals are present in the form of kaolinite as a major constituent in Local Selveia, Hashmi rock, and Golden Sinai samples and as a minor constituent in Fass Galala and Gandola granite.

(d) Quartz: Infrared analysis reveals only a moderate presence of quartz in Golden Sinai, limestone, Gondola granite, and Red Royal granite samples. 

(e) Feldspars: Examination of the obtained spectra illustrates the abundance of albite in the two types of granite, i.e., Gondola and Red Royal and in the Indian Green marble. Albite is rarely present in Hashmi rock and completely absent in other types. Microcline can be detected also but, only in Gondola granite and Red Royal granite. 

(f) Alkanes: The presence of organic compounds as alkanes is evident through examining the infrared spectra of the investigated rock samples, where alkanes are present in considerable amounts in all samples except in the two types of granite (Gondola and Royal) and Hashmi rock. According to [1], the presence of alkanes in rocks is due to the precipitation of the fauna in these rocks during their formation in ancient era. The organic compounds were transformed to CO_2_ and water through the combustion process. This combustion was replaced by bacteriological decay or direct oxidation. It appears that based on the amount of oxygen available, the organic residues were exposed to partial oxidation. This incomplete or partial oxidation is known as putrefaction [35].

### 3.3. X-ray Diffraction Analysis

#### 3.3.1. Dust Fall Samples

**Figure 6:**
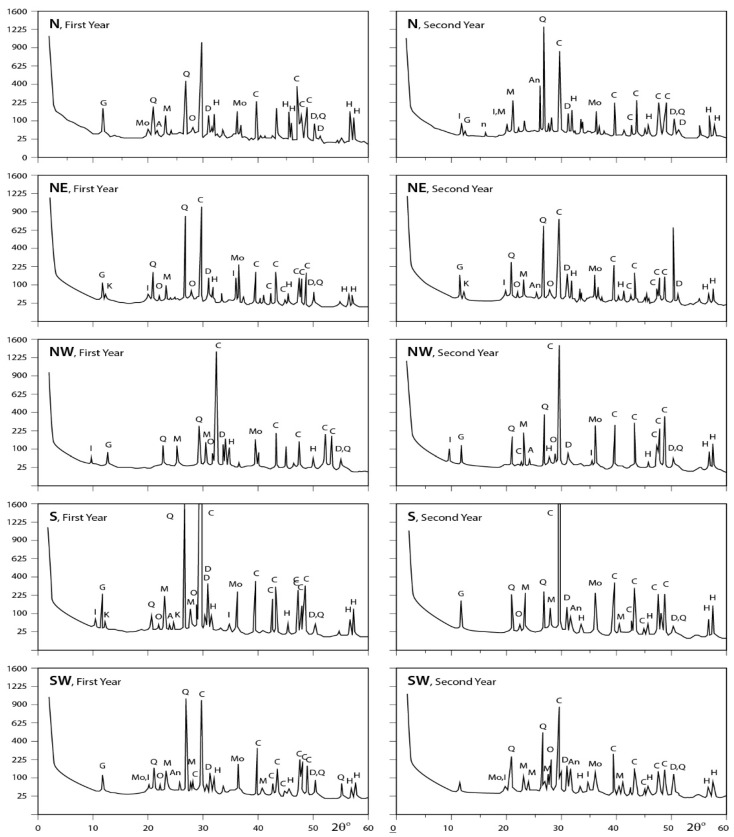
XRD profiles for the analyzed samples; A = albite; An = anhydrite; At = antigorite; C = calcite; D = dolomite; F = feldspar; G = gypsum; H = halite; I = illite; K = kaolinite; M = microcline; Mo = montmorilonite; Ms = muscovite; O = orthoclase; Q = quartz; T = tremolite (N: North; NW: Northwest; NE: Northeast; S: South; SW: Southwest).

**Figure 7:**
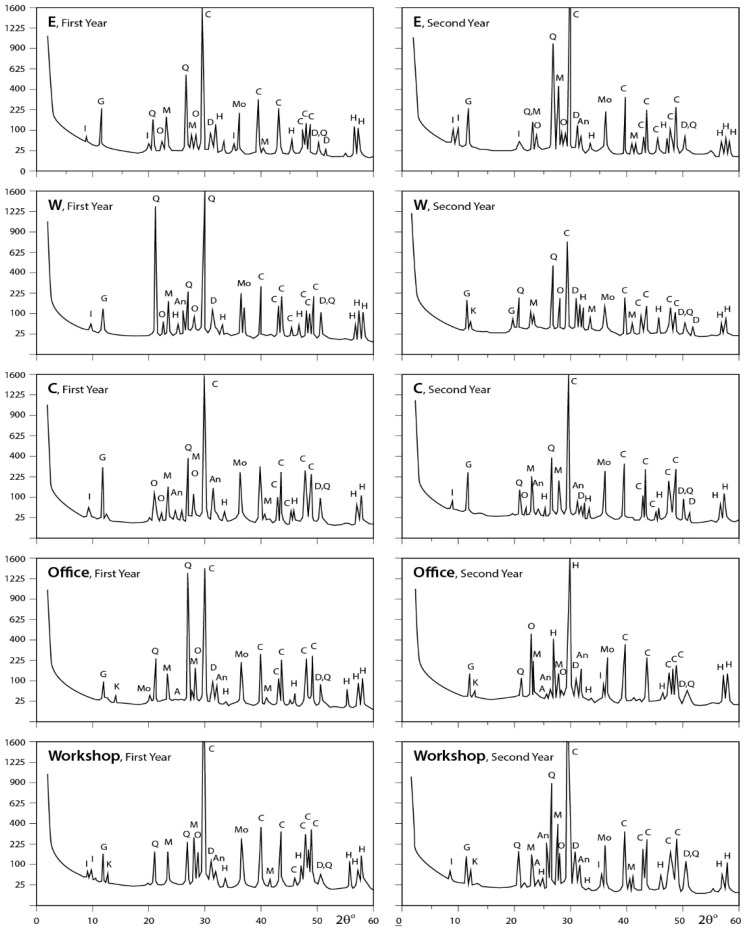
XRD profiles for the analyzed samples. A = albite; An = anhydrite; At = antigorite; C = calcite; D = dolomite; F = feldspar; G = gypsum; H = halite; I = illite; K = kaolinite; M = microcline; Mo = montmorilonite; Ms = muscovite; O = orthoclase; Q = quartz; T = tremolite (E, W, and C represent the different locations).

The obtained X-ray diffraction patterns for the dust fall samples collected from the different sites are presented in Figure 5, Figure 6 and Figure 7 and the diffraction data are given in the same figures. These patterns reveal that calcite is the major mineral constituent of dust fall in the study area. Quartz is less than calcite in abundance. However, it still is one of the major mineral constituents of the dust fall in all study locations—except the northwest, center, and workshop locations, where it is present as a minor mineral. Gypsum is present as a minor constituent in all locations of study. Anhydrite appears in trace amounts in all studied locations, meanwhile dolomite is present in minor quantities overall the study area, but it is concentrated in south and southwest locations. Traces of feldspars, kaolinite, illite, montmorillonite, and halite are recorded in the studied sites.

#### 3.3.2. Rock Samples

**Figure 8:**
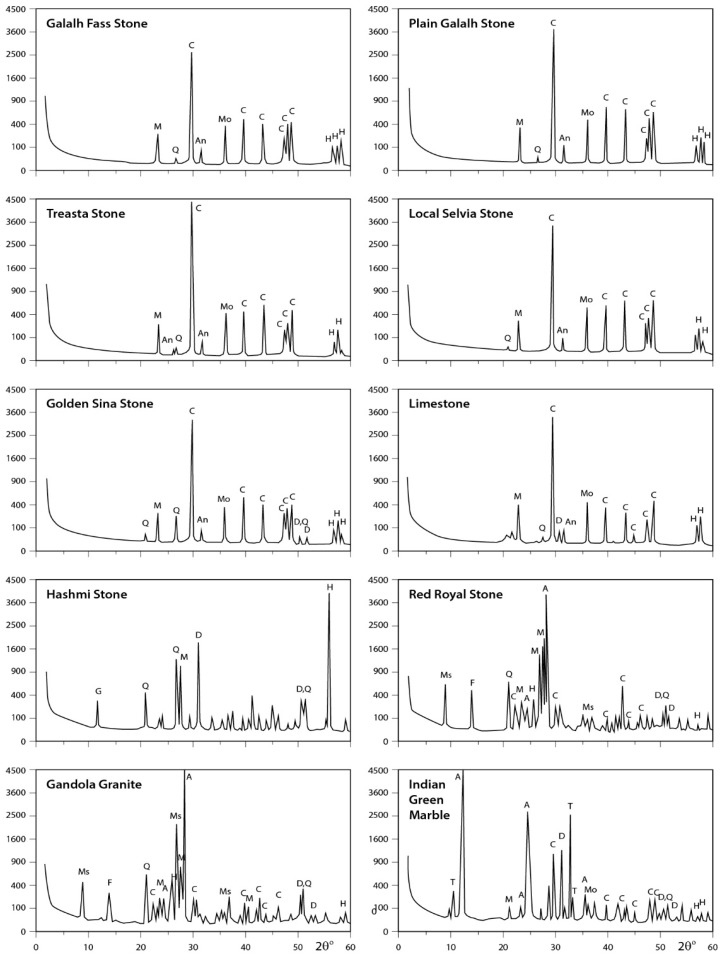
XRD profiles for the analyzed samples. The identified minerals are: A = albite; An = anhydrite; At = antigorite; C = calcite; D = dolomite; F = feldspar; G = gypsum; H = halite; I = illite; K = kaolinite; M = microcline; Mo = montmorillonite; Ms = muscovite; O = orthoclase; Q = quartz; T = tremolite.

X-ray data for the analyzed rocks are illustrated in Figure 6, Figure 7 and Figure 8. The obtained results indicate that Fass Galala, Plain Galala, Treasta, Local Selveia, Golden Sinai, and limestone have the same main mineral composition (calcite in majority; quartz in minority; and traces of halite, montmorillonite, and anhydrite), however Golden Sinai and limestone contains traces of dolomite in addition to this composition. Hashmi rock contains considerable amounts of dolomite and halite and traces of gypsum, quartz, and microcline. The two granite samples analyzed (Gondola and Red Royal) have the same composition (albite in majority; muscovite in minority; and traces of calcite, dolomite, quartz, feldspar, anhydrite, and halite). Indian Green marble is mainly composed of antigorite; minor amount of tremolite; and traces of feldspar, calcite, dolomite, montmorillonite, and halite.

Mineralogical investigation of the dust fall samples in Shaq El−Teeban industrial area by means of the three techniques applied—i.e., polarizing microscope, Infrared spectroscopy, and X-ray diffraction analysis—reveals that they are mainly composed from the following minerals:

(a) Carbonates: Carbonates are the main component of dust fall over the study area. This finding can be correlated with the mineral predominance in the rocks that are processed in this industrial area. It is present mainly in calcite form (CaCO_3_), meanwhile dolomite (CaMg(CO_3_)_2_) appears in trace amounts in dust fall overall most of the study area and concentrated in only two sites (south and southwest). This is due to workshops cutting dolomite-rich rocks (Hashmi rock) being concentrated in the southern sector of the area under consideration.

(b) Quartz: Quartz is the second-most abundant mineral after calcite in the atmosphere of the study area where it is present in major quantities in all sites except the southwest, center, and workshop sites where it is present in minor amounts.

(c) Sulphates: Sulphates are mostly present in the gypsum form (CaSO_4_·2H_2_O) and distributed in minor quantities in dust fall over all sites—except northwest, southwest, office, and workshop locations where it is present in trace amounts. Sulphates are also present in the anhydrite form (CaSO_4_) as traces in dust fall overall the study area.

(d) Other Traces: Feldspars (albite, microcline, and orthoclase), clay minerals (kaolinite, illite, and montmorillonite), and halite are present in dust fall in the study area as traces. Alkanes are present in considerable amounts in dust fall over all sites in the study area. Meanwhile, phosphines are present in trace amounts in five locations only, i.e., the north, southwest, east, center, and workshop locations. 

The identified minerals and organic compounds observed in the dust fall over the area of interest and their relative abundances are concordant with those observed in the investigated rocks and nearly have the same relative abundance. This indicates that the main source of particulate pollution in the study area is the activities taking place in the area that entrap the dust and the observed chemicals. These activities are associated with the processing of these rocks which are common in the study area. However, a minor contribution comes from the natural sources by wind effects, both as an erosion tool and as a carrier of dust, especially from Tura and Mokkatam hills.

Alkanes are mainly considered as a man−made pollutant. The major source of these organic compounds is the combustion of fuels such as gasoline, diesel, and to a lesser extent wood. The alkanes are produced during incomplete combustion of these fuels where traces of paraffin are emitted, unchanged or only partially cracked, into the atmosphere. Besides, it has been reported that the limestones and other carbonate rocks are responsible, among other known sources, for the buildup of aliphatic hydrocarbons in airborne particulate matter in nearby areas [1]. This explains the considerable presence of alkanes in the atmosphere of the area under investigation where the processing of carbonate rocks, especially limestone, predominates.

Phosphines which are organic phosphorous compounds are commonly adsorbed on clay minerals [36]. They are usually produced through the anaerobic bacterial action on the organic deposits. Throughout the two years of study, the obtained results show that the qualitative mineralogical analysis of solid air pollutants does not differ greatly from one year to another.

The obtained results from the three techniques applied in the present study are nearly similar. The slight differences between the three types of results are due to the difference in nature and powerful characteristics of each technique and the presence of the investigated components sometimes in trace amounts, for example the absence of hornblende in XRD and IR analysis and its appearance in microscopic examination and the absence of halite and anhydrite in microscopic examination and IR analysis and its appearance in XRD analysis.

## 4. Conclusions

The obtained results provide information on the contribution of rock processing activities to the mineral composition of atmospheric particulates. The presented data make a strong point to differentiate between pure chemical analysis and mineralogical analysis; since pollution and health hazards are not the same for chemicals and minerals. However, the major mineralogical species existing in the atmosphere of southeast Cairo are present in the following order: calcite > quartz > gypsum > dolomite > anhydrite, feldspars, clay minerals in the form of kaolinite, illite, and montmorillonite, and halites. Organic compounds are present in the atmosphere of the area mainly in the form of alkanes (saturated aliphatic hydrocarbons) with presence of traces of phosphines (organic phosphorous compounds). The main source of airborne minerals and associated organic compounds in the study area is the industrial activities, mainly rock processing with minor contributions from natural sources—especially erosion and dust carried by winds from Tura and Mokkatam hills—which are surrounding Cairo from the southern direction. The obtained results from the microscopic examination, infrared spectroscopy, and X-ray diffraction analysis are nearly similar and the observed few differences are due to the nature and powerful characteristics of each technique used and the presence of some investigated components in trace amounts. 

## Figures and Tables

**Table 1:** Infrared spectrum frequencies of common minerals.

Mineral	Formula	Spectrum Frequencies		
**1−Carbonates**				
Calcite	CaCO_3_	2517	1798	1435(s)
		873(m)	712(m)	
Dolomite	Ca Mg(CO_3_)_2_	1435(s)	881(m)	730
**2−Sulphate**				
Gypsum	CaSO_4_	3410(m)	1626(m)	1142(s)
		1114(s)	1004(m)	670(m)
		600§		
**3−Oxides**				
Magnetite	Fe_3_O_4_	575(b,w)		
Hematite	Fe_2_O_3_	550§	475	
Ilmenite	FeTiO_3_	700(w)	540(s,b)	455
**4−Silicon oxides**				
Quartz	SiO_2_	1163(s)	1078(s)	798§(m)
Amorphous silica	SiO_2_	779§(m)	695(m)	455
		1700		
**5−Silicates**				
A−Clay minerals				
Kaolinite	Al_2_Si_2_O_5_(OH)_4_	3705	3673(w)	3663(m)
		3613	3547	1667(w)
		1105(s)	1031§(s)	1006(s)
		935§(m)	909§(s)	545
		(& others)		
Montmorillonite	(Al,Mg)_2_Si_4_O_10_(OH)_2_.nH_2_O	3571(m)	1626(m)	1117(m
		1042(s)	909(m)	(& others).
Illite	K_2−3_ Al_11_Si_12_O_35−36_	1639(w)	1117(m)	1031(s)
		909§(m)	(& others)	
B−Feldspars				
Albite	NaAlSi_3_O_8_	1134(s)	1083(s)	1028
		1015(s)	986	758(m)
		645§(m)	529§	462
		427		
Orthoclase	KAlSi_3_O_8_	641(b)		
Microcline	KAlSi_3_O_8_	1128(s)	1089(s)	1037
		1009(s)	935	792
		768(m)	726(m)	648§(w)
		606	585	533§
		427		
C−Mica				
Biotite	K(Mg,Fe)_3_(AlSi_3_O_10_)(OH)_2_	1067	988	770
		745	660	630
		449		

Note: w = weak; m = medium; s = strong; b = broad; § = diagnostic peak.

**Table 2:** Infrared spectrum frequencies of some organic compounds.

Organic Compound	Formula	Spectrum Frequency
1−Alkanes	CnH_2n+2_	2982
		2925 ± 10(s) asym. Stretch
		2850 ± 10(s) sym. Stretch
		1465 ± 20(m) asym. Bending
2−Phosphines	R_2_PH	2440–2350(m) P–H stretchsharp absorption band.
		1450–1435(m) P–R stretch, where R = aryl.
		1320–1280(m) P–R stretch,where R = CH_3_ only.
3−Silicon hydrides	Si–H	2280–2080(s) Si–H stretch

Note: m = medium; s = strong.
